# Comparative analysis of commonly used peak calling programs for ChIP-Seq analysis

**DOI:** 10.5808/GI.2020.18.4.e42

**Published:** 2020-12-14

**Authors:** Hyeongrin Jeon, Hyunji Lee, Byunghee Kang, Insoon Jang, Tae-Young Roh

**Affiliations:** 1 Department of Life Sciences, Pohang University of Science and Technology (POSTECH), Pohang 37673, Korea; 2 Division of Integrative Biosciences and Biotechnology, Pohang University of Science and Technology (POSTECH), Pohang 37673, Korea; 3 SysGenLab Inc., Pohang 37613, Korea

**Keywords:** ChIP-Seq, histone modification, human embryonic stem cell, peak calling program

## Abstract

Chromatin immunoprecipitation coupled with high-throughput DNA sequencing (ChIP-Seq) is a powerful technology to profile the location of proteins of interest on a whole-genome scale. To identify the enrichment location of proteins, many programs and algorithms have been proposed. However, none of the commonly used peak calling programs could accurately explain the binding features of target proteins detected by ChIP-Seq. Here, publicly available data on 12 histone modifications, including H3K4ac/me1/me2/me3, H3K9ac/me3, H3K27ac/me3, H3K36me3, H3K56ac, and H3K79me1/me2, generated from a human embryonic stem cell line (H1), were profiled with five peak callers (CisGenome, MACS1, MACS2, PeakSeq, and SISSRs). The performance of the peak calling programs was compared in terms of reproducibility between replicates, examination of enriched regions to variable sequencing depths, the specificity-to-noise signal, and sensitivity of peak prediction. There were no major differences among peak callers when analyzing point source histone modifications. The peak calling results from histone modifications with low fidelity, such as H3K4ac, H3K56ac, and H3K79me1/me2, showed low performance in all parameters, which indicates that their peak positions might not be located accurately. Our comparative results could provide a helpful guide to choose a suitable peak calling program for specific histone modifications.

## Introduction

Protein-binding regions in the context of chromatin have been detected by the chromatin immunoprecipitation (ChIP) method. Since the first ChIP coupled with high-throughput DNA sequencing (ChIPSeq) technology for histone modification mapping was introduced with the combination of ChIP and next-generation sequencing, a large amount of ChIP-Seq data has been produced at the genome level, and the development of data analysis tools should thus be emphasized [1-3].

The basic building block of chromatin, the nucleosome, consists of 146 base pairs (bp) of DNA and a histone octamer composed of four core histones: H2A, H2B, H3, and H4. Post-translational modifications of histone tails play an important role in the epigenetic regulation of genome activity. These modifications include acetylation, methylation, phosphorylation, and ubiquitination. Depending on the types of histone modifications and binding sites, different enrichment patterns and related biological effects are expected. For example, acetylated histones provide a chromatin environment easily accessible to the transcriptional machinery by changing the chromatin conformation. Some histone methylations, such as H3K4me2 and H3K4me3, are mostly located on promoters, whereas H3K36me3 is predominantly found on the gene bodies of transcriptionally active genes [4,5].

The Encyclopedia of DNA Elements (ENCODE) Consortium, aiming at the identification of all functional elements in the human genome, proposed a guideline for categorizing protein-bound regions occupied by point source factors, broad source factors, and mixed source factors [6].

The distribution patterns of ChIP-Seq data on the genome have been analyzed using many different software programs with specific algorithms, which use different strategies for searching potential binding regions, judging the peaks, and calculating significance [7-10]. Most previous studies have focused on detecting the enriched peaks, and several groups have already evaluated peak calling programs [11-16]. Although most previous studies compared the performance of each program for analyzing transcription factor binding patterns, some tested histone modifications, including H3K4me3, H3K9me3, H3K27me3, and H3K36me3 [11,12,14]. However, the performance evaluation of ChIP-Seq analysis programs needs to be more extensively examined to understand the nature of enrichment of various types of histone modifications. Herein, we tested ChIP-Seq data from 12 histone modifications covering three source types with five peak calling programs (CisGenome, MACS1, MACS2, PeakSeq, and SISSRs).

## Methods

### Data filtering and cross-correlation analysis

The ChIP-Seq datasets of 12 histone modification types, input, and RNA-sequencing of human embryonic stem cell line (H1) were downloaded from the NIH Roadmap Epigenomics Project Gene Expression Omnibus (GEO) repository (http://www.ncbi.nlm.nih.gov/geo/roadmap/epigenomics/) (Supplementary Table 1). The downloaded SRA format files were converted to the FASTQ format via fastq-dump in SRA Toolkit (version 2.4.5). Raw sequencing reads were filtered by fastq_quality_filter (FASTX-Toolkit version 0.0.13.2) with the following options (-p 80, -q 20, and -Q33). High-quality reads were mapped to the human genome (hg19) using Bowtie (version 1.1.1) with the default options (-n 2, -e 70, -l 28, -I 0, -X 250, and -maxbts 250) [17].

To evaluate the signal-to-noise ratio of a ChIP-Seq experiment, strand cross-correlation analysis was performed using the SPP program with the default options (-s -100:5:600, and -x 10), considering two metrics: (1) the normalized strand coefficient, which quantifies the fragment length cross-correlation over the background cross-correlation rate, and (2) the relative strand correlation, which calculates the ratio of cross-correlation observed at the predicted fragment size against the artifactual cross-correlation observed at the read length [18].

### Identification of regions enriched with specific histone modifications

To detect peaks, CisGenome (version 2.0), MACS1 (version 1.4.2), MACS2 (version 2.1.0), PeakSeq (version 1.31), and SISSRs (version 1.4), were used with the default options and recommended parameters for a direct comparison without any optimization (Supplementary Table 2). For CisGenome, the Bowtie-format output files were converted into the aln format and the seqpeak command was used. For MACS1, the options of –p 1e-5, -m 10:30, and --keep-dup 1 were used and for MACS2, the default options (-q 0.01, -m 5:50, and --keep dup 1) were applied. In MACS2, the broad options (-q 0.1, -m 5:50, and --keep-dup 1) were also used for the broad source peaks. The signal map was prepared from the Bowtie output using the PeakSeq -preprocess command. During the step of PeakSeq -peak_selection, the default options were used, such as Enrichment_mapped_fragment_length 200, target_FDR 0.05, N_Simulations 10, Minimum_interpeak_distance 200, and max_Qvalue 0.05. SISSRs detected peaks with the recommend options (-F 0.001, -e 10, -p 0.001, -m 0.8, -w 20, -E 2, and -L 500). All peaks in each set were ranked by the following guidelines: CisGenome and PeakSeq, pre-sorted peak lists; MACS1 and MACS2, sorted by the significance level (10 × 2log10(p-value)) and then by the fold enrichment; SISSRs, ranked by the fold enrichment and by the significance level (p-value). Frequently detected false positive peaks, regardless of cell line or experiment (called the ENCODE blacklist) were removed for quality control of peaks [19,20].

### Comparison of peak calling performance

The coincidence of peak positions obtained by the individual programs was examined using the intersectBed and multiIntersectBed functions (BEDTools version 2.23.0) with a minimum overlapping size of 1 bp [21]. Pearson correlation coefficients based on peak ranks between overlapped peaks were calculated, because the peak rank represents the order of importance according to algorithm characteristics. For the multiple comparison analyses of each histone mark, we used multiIntersectBed in BEDTools. The multiIntersectBed function provided a comparison among the multiple files.

The Jaccard similarity coefficients (or index J) were calculated for the measurement of variability: J(A, B) = |A ∩ B| / |A∪B| where A and B are sets of enriched regions in base pairs identified by peak calling programs. Irreproducibility discovery rate (IDR) analysis with all replicates was performed using the recommended parameters (peak.half.width ‒1, min.overlap.ratio 0, is.broadpeak F, and ranking.measure p.value for MACS1 and MACS2; q.value for CisGenome and PeakSeq; signal.value for SISSRs) [22]. For the specificity test, the control sequence reads were mixed with the original ChIP-Seq data and then the performance was computed. At a different sequencing read depth, the genomic coverage of the enriched regions was calculated by genomeCoverageBed in BEDTools.

The genomic coverage of the regions was calculated by genomeCoverageBed in BEDTools by considering randomly selected reads (0.5, 0.75, 1.0, 2.5, 5.0, 7.5, 10, 15, 20, and 30 million). To detect enriched regions in subsampled data, the algorithms with the same parameters as in the above analysis were used. The specificity of the immunoprecipitated signals to nonspecific noise was examined by mixing the ChIP-Seq data with different noise levels (50% 100%, and 150% of control reads).

## Results

### Overview of ChIP-Seq data analysis

**Fig. 1.**
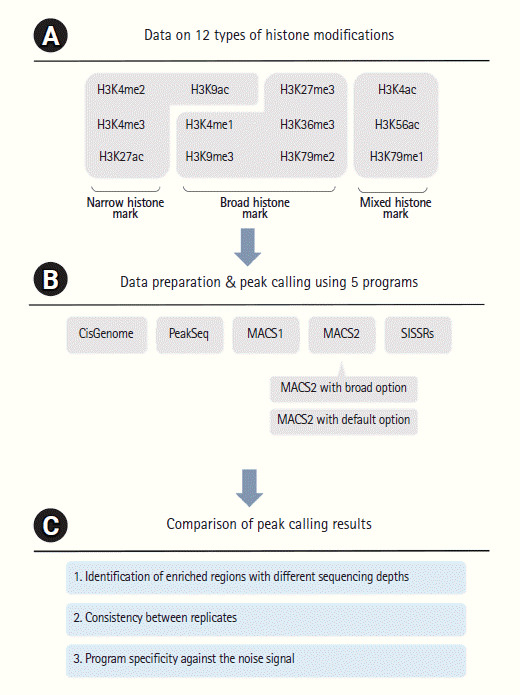
Overview of the analysis. (A) Histone modifications are classified as narrow, broad, and mixed types. (B) Five programs were used for data preparation and peak calling. MACS2 was executed with the default or broad option. (C) The called peaks were compared in terms of enrichment, consistency, and specificity.

For the comparative analysis of ChIP-Seq peak calling programs, data on 12 types of histone modifications were initially filtered and only high-quality mappable reads were used for further analysis (Supplementary Table 3). The histone modification marks were grouped into narrow (4 histone modifications), broad (5), and mixed (3) sources according to the ENCODE guideline. Peaks were called by five commonly used programs [7-10] and their number, position, coverage, and specificity were compared individually. An overview of this study is summarized in Fig. 1.

### Concordance of peak regions

**Fig. 2.**
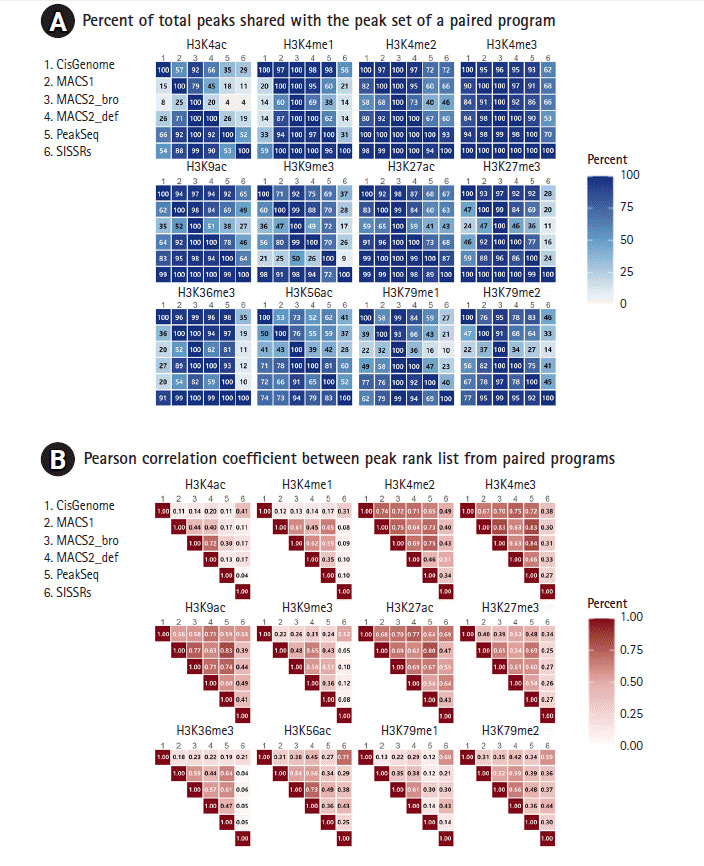
Pairwise comparison of shared regions. (A) Percentage of peaks recaptured by programs shown pairwise. Each panel shows the percentage of total peaks from one method (column) that was recaptured by another peak caller (row) after filtering blacklist peaks. (B) The concordance rate of peak regions derived from two peak callers. The ranked coincidence weas calculated and the values of percentage and correlation coefficients were denoted after filtering blacklist peaks.

The peaks representing the enrichment patterns of each histone modification were more affected by histone types than by peak calling programs. The peak counts of H3K4me3, H3K9ac, H3K27me3, and H3K56ac were similar in most peak calling programs except SISSRs. Peak lengths were strongly affected by the program used, with the average length varying from 57.7 to 1941.8 bp (Supplementary Table 4). Peaks from MACS2 with the broad option and PeakSeq covered a longer genomic region, while CisGenome, MACS1, MACS2 with the default, and SISSRs suggested relatively short regions as peaks. Notably, SISSRs identified the shortest peaks. The concordance or co-occupancy of peaks regions identified from two different callers were calculated at the same genomic loci. The peaks from H3K4me2, H3K4me3, H3K9ac, H3K27me3, and H3K36me3 varied in length. As a representative example, the number of peaks enriched with H3K4me3, a typical narrow source mark, ranged from 24,000 to 37,000 and its enrichment profile was very similar at promoters of actively transcribed genes with all peak callers (Fig. 2A). The peak positional variability was highly dependent on the histone mark type. Histone marks such as H3K4me2, H3K4me3, H3K27ac, and H3K9ac, which are associated with transcriptional activation, showed a high level of concordance. The overlapping ratio of H3K4ac and H3K79me1 was below 60% on average. Our results indicated that histone marks that covered narrow regions with high enrichment could be identified by any of the peak callers used in this study, but peak positions from broad source marks differed according to the peak calling algorithm.

The significance of the identified peaks was examined by Pearson correlation coefficients. To explore peak coherence among algorithms, the correlation coefficients of histone types were categorized (Fig. 2B). The highest correlations were obtained from the peaks of H3K4me2, H3K4me3, H3K9ac, and H3K27ac. The lowest group included H3K4ac, H3K4me1, H3K9me3, H3K36me3, and H3K79me1.

### Peak consistency between replicates

**Fig. 3.**
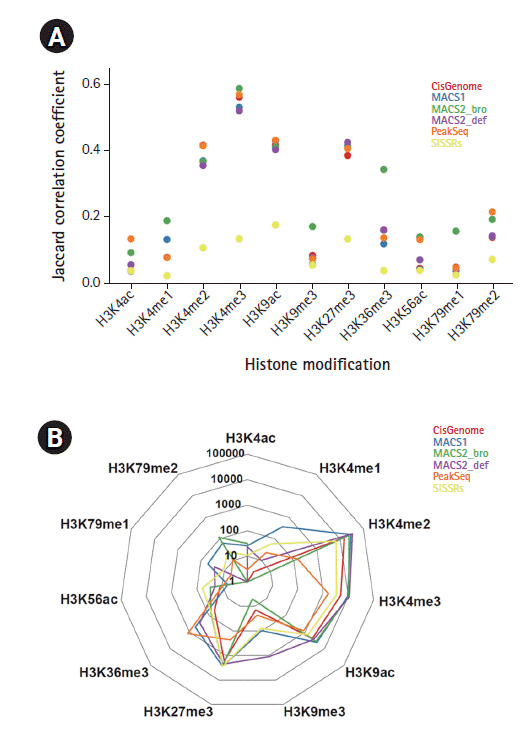
Peak consistency between replicates (A) Jaccard correlation coefficient between biological duplicates for each histone ChIP-Seq data. (B) Reproducible peak numbers passing the IDR threshold of 0.01%.

The reproducibility of the peak calling algorithm across biological replicates was measured by considering the Jaccard similarity and the IDR [22]. The Jaccard similarity coefficients between replicates at a single base level were computed except for H3K27ac, for which the duplicated data set was not provided. H3K4me2, H3K4me3, H3K9ac, and H3K27me3 had high similarity between replicates in all peak callers except SISSRs (Fig. 3A). The mean values of the Jaccard similarity coefficients between H3K4me3 replicates were above 0.5. Interestingly, the similarity between H3K36me3 replicates was higher only in MACS2 with the broad option than in any other callers, which means that H3K36me3 mark clearly belongs to the group of broad source marks. The consistency of other histone modifications was fairly low. Some significant histone type-dependent consistency was detected, such as for H3K4me1, H3K9me3, and H3K79me1 with MACS2 with the broad option; H3K4ac and H3K79me2 with PeakSeq; and H3K56ac with MACS2 with the broad option and PeakSeq.

According to the ENCODE guidelines [6], the IDR should be used for narrow peaks such as transcription factors, as well as for punctate chromatin marks such as H3K4me1, H3K4me3, H3K9ac, and H3K27ac. Considering the average number of peaks reducible in replicate pairs with an IDR threshold of 0.01%, the reproducibility of different peak callers was dependent on the histone type (Fig. 3B). H3K4me2 and H3K4me3 showed a relatively large number of reproducible peaks. The MACS1 program gave the most reproducible results across replicates in these histone modifications. The peaks identified from H3K4ac, H3K56ac, H3K79me1, and H3K79me2 seemed not to be reproducible.

### Peak coverage with different sequencing depths

**Fig. 4.**
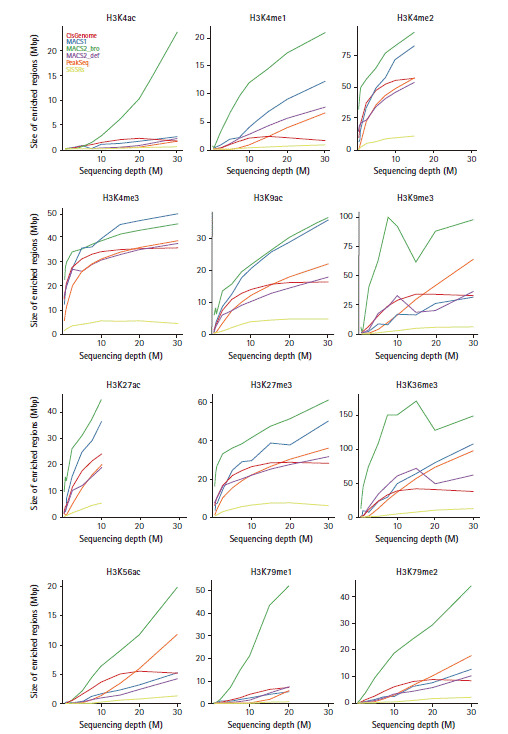
Peak coverage with different sequencing depths. The genomic coverage of the regions was shown by sampling with different sequence reads (0.5, 0.75, 1.0, 2.5, 5.0, 7.5, 10, 15, 20, and 30 million).

The importance of sequencing depth has been emphasized for measuring the experimental validity of ChIP-Seq. To assess the number of peaks at the level of sequencing read saturation by different peak calling algorithms, the peak calling procedure was repeatedly applied with different numbers of subsampled reads from the total number of sequencing reads (Fig. 4). The peak coverage of point source marks in all peak callers except SISSRs dramatically increased at a lower depth (≤2.5 million reads for H3K4me2 and ≤1 million reads for H3K4me3). Broad source marks like H3K9ac, H3K27ac, H3K27me3, and H3K36me3 needed more reads to reach the level of saturation and their coverage of enriched genomic regions was consistently increased at >10 million reads. The size of enriched regions derived from MACS2 with a broad option generally covered larger loci than any other algorithms.

### Specificity of peak calling against the noise signal

**Fig. 5.**
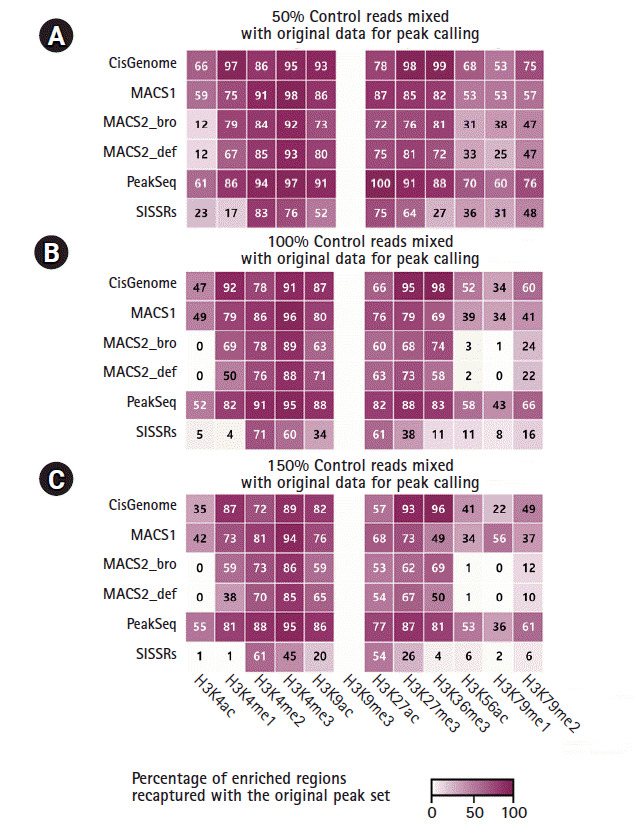
Specificity of peak calling against the noise signal. The specificity of each program was calculated by sampling with different noise levels. Fifty percent (A), 100% (B), and 150% (C) of control reads added.

The peak specificity called by different algorithms was compared by mixing the ChIP-Seq reads with randomly-selected input control reads (50%, 100%, and 150% of the corresponding ChIP-Seq reads). The percentage of enriched regions recaptured by CisGenome, MACS1, and PeakSeq was not substantially affected by the noise level (Fig. 5). CisGenome and PeakSeq recaptured over 80% of the enriched regions even with noise reads for H3K4me1, H3K4me3, H3K9ac, H3K27me3, and H3K36me3. In particular, MACS2 was very responsive to the noise and the recaptured peak ratio fell down to the minimum level when it was tested with the H3K4ac, H3K56ac, and H3K79me1 marks. SISSRs had the lowest performance for peak recapturing. Interestingly, the recaptured peak ratio of the H3K9me3 mark was dramatically decreased with all peak callers, which implied that the sequencing depth and the number of peaks for this modification might not reach the saturation level.

## Discussion

The identification of exact protein-binding sites on chromatin is the most important step for ChIP-Seq analysis. Many ChIP-Seq peak calling programs and algorithms have been published. Some of them compared individual performance for transcription factor binding profiles. In this study, to obtain relevant information for the practical usage of peak callers, we analyzed the enrichment of 12 histone marks at specific genomic regions with respect to different sequencing depths, consistency between replicates, specificity, and correlation. Generally, narrow source histone modifications like H3K4me2, H3K4me3, H3K9ac, and H3K27ac showed relatively consistent peaks across the peak callers. However, the peaks identified from H3K4ac, H3K56ac, H3K79me1, and H3K79me2 ChIP-Seq data varied depending on the peak caller, which means that the proper choice of a peak caller is critical.

For the evaluation of reproducibility of peak detection, the Jaccard similarity coefficient and the IDR were considered and both gave fairly good results with point source marks. The broad source marks had lower Jaccard correlation coefficients and low reproducibility.

The sequencing depth, or the count of sequencing reads, is an important factor for identifying the region occupied by a specific protein factor in the genome. A recent study suggested that a sufficient sequencing depth for human ChIP-Seq is 40‒50 million reads [9]. However, most published ChIP-Seq data did not reach this read count, probably due to the sequencing cost. Considering sequencing depth, we also analyzed the effect of peak calling performance with different numbers of sequencing reads. The size distribution of enriched regions occupied by peaks was saturated under 1 million reads for H3K4me3 and 2.5 million reads for H3K4me2, but most broad source marks like H3K4me1, H3K9me3, H3K27me3, H3K36me3, and H3K79me2 did not show a distinct saturation profile due to the low sequencing depth or a histone modification type-specific feature.

The validity of ChIP-Seq data can be assessed by the specificity of peaks distinguishing true peaks from noise. Randomly selected noise reads from ChIP-Seq input data were used to test the specificity. The individual peak callers showed a good performance with 50% of noise reads, but with a high amount of noise signal, only two callers (CisGenome and PeakSeq) could recapture the original peak regions consistently.

The purpose of this comparative study was to provide practical suggestions for the selection of ChIP-Seq peak calling programs, and thus the comparison of the algorithms and/or statistics used in each program was beyond our research scope. Our results indicated that a proper selection of the peak caller considering the histone modification type is a critical step for the identification of protein-enriched regions specifically. In particular, the peaks occupied by broad and mixed histone marks were dramatically affected by the performance of the peak caller. Based on this study, we constructed an optimal analysis pipeline for ChIP-Seq data and have provided a free ChIP-Seq analysis tool at the Korean Bioinformation Center (KOBIC) (https://closha.kobic.re.kr/).

## Supplementary Materials

Supplementary Table 1. List of ChIP-Seq data

Supplementary Table 2. Peak calling programs

Supplementary Table 3. Alignment summary

Supplementary Table 4. Average peak length (bp)
