# Supplementary material for: Comparative analysis of commonly used peak calling programs for ChIP-Seq analysis
Source: Genomics Inform. 2020 Dec 14;18(4):e42. doi: 10.5808/GI.2020.18.4.e42 (PMC7808876; doi:10.5808/GI.2020.18.4.e42)
Supplement: Supplementary Table 1. — List of ChIP-Seq data [file gi-2020-18-4-e42-suppl1.pdf]

Supplementary Table 1. List of ChIP-Seq data

GEO accession	SRA	Cell type	Factor	ID
GSM605311	SRR067946	H1 cell line	H3K4ac	H1_H3K4ac_1_1
GSM667624	SRR179735	H1 cell line	H3K4ac	H1_H3K4ac_2_1
GSM667624	SRR179736	H1 cell line	H3K4ac	H1_H3K4ac_2_2
GSM667624	SRR179737	H1 cell line	H3K4ac	H1_H3K4ac_2_3
GSM409307	SRR018456	H1 cell line	H3K4me1	H1_H3K4me1_1_1
GSM433177	SRR020519	H1 cell line	H3K4me1	H1_H3K4me1_2_1
GSM434762	SRR019560	H1 cell line	H3K4me1	H1_H3K4me1_3_1
GSM466739	SRR029339	H1 cell line	H3K4me1	H1_H3K4me1_4_1
GSM466739	SRR029615	H1 cell line	H3K4me1	H1_H3K4me1_4_2
GSM466739	SRR029619	H1 cell line	H3K4me1	H1_H3K4me1_4_3
GSM537679	SRR1068412	H1 cell line	H3K4me1	H1_H3K4me1_5_1
GSM605312	SRR067947	H1 cell line	H3K4me1	H1_H3K4me1_6_1
GSM602260	SRR067768	H1 cell line	H3K4me2	H1_H3K4me2_1_1*
GSM602260	SRR067948	H1 cell line	H3K4me2	H1_H3K4me2_1_2
GSM602261	SRR067769	H1 cell line	H3K4me2	H1_H3K4me2_2_1
GSM409308	SRR018455	H1 cell line	H3K4me3	H1_H3K4me3_1_1
GSM410808	SRR018232	H1 cell line	H3K4me3	H1_H3K4me3_2_1
GSM432392	SRR019222	H1 cell line	H3K4me3	H1_H3K4me3_3_1
GSM433170	SRR020515	H1 cell line	H3K4me3	H1_H3K4me3_4_1*
GSM469971	SRR029609	H1 cell line	H3K4me3	H1_H3K4me3_5_1*
GSM469971	SRR029620	H1 cell line	H3K4me3	H1_H3K4me3_5_2
GSM537680	SRR1068413	H1 cell line	H3K4me3	H1_H3K4me3_6_1*
GSM537681	SRR1068414	H1 cell line	H3K4me3	H1_H3K4me3_7_1
GSM605315	SRR067950	H1 cell line	H3K4me3	H1_H3K4me3_8_1
GSM410807	SRR018231	H1 cell line	H3K9ac	H1_H3K9ac_1_1
GSM433171	SRR020516	H1 cell line	H3K9ac	H1_H3K9ac_2_1
GSM434785	SRR019562	H1 cell line	H3K9ac	H1_H3K9ac_3_1
GSM537685	SRR1068418	H1 cell line	H3K9ac	H1_H3K9ac_4_1
GSM605323	SRR067958	H1 cell line	H3K9ac	H1_H3K9ac_5_1
GSM428291	SRR018478	H1 cell line	H3K9me3	H1_H3K9me3_1_1
GSM428291	SRR018479	H1 cell line	H3K9me3	H1_H3K9me3_1_2
GSM428291	SRR018480	H1 cell line	H3K9me3	H1_H3K9me3_1_3
GSM428291	SRR018481	H1 cell line	H3K9me3	H1_H3K9me3_1_4
GSM433174	SRR020517	H1 cell line	H3K9me3	H1_H3K9me3_2_1
GSM450266	SRR026664	H1 cell line	H3K9me3	H1_H3K9me3_3_1
GSM450266	SRR026665	H1 cell line	H3K9me3	H1_H3K9me3_3_2

GSM605325	SRR018453	H1 cell line	H3K9me3	H1_H3K9me3_4_1
GSM605325	SRR067960	H1 cell line	H3K9me3	H1_H3K9me3_4_2
GSM605325	SRR067961	H1 cell line	H3K9me3	H1_H3K9me3_4_3
GSM605325	SRR067962	H1 cell line	H3K9me3	H1_H3K9me3_4_4
GSM605325	SRR067963	H1 cell line	H3K9me3	H1_H3K9me3_4_5
GSM605327	SRR067964	H1 cell line	H3K9me3	H1_H3K9me3_5_1
GSM663427	SRR179707	H1 cell line	H3K27ac	H1_H3K27ac
GSM1185386	SRR933992	H1 cell line	H3K27me3	H1_H3K27me3_1_1
GSM1185386	SRR933993	H1 cell line	H3K27me3	H1_H3K27me3_1_2
GSM428295	SRR018226	H1 cell line	H3K27me3	H1_H3K27me3_2_1
GSM428295	SRR018477	H1 cell line	H3K27me3	H1_H3K27me3_2_2
GSM433167	SRR020508	H1 cell line	H3K27me3	H1_H3K27me3_3_1*
GSM434776	SRR019561	H1 cell line	H3K27me3	H1_H3K27me3_4_1
GSM466734	SRR029343	H1 cell line	H3K27me3	H1_H3K27me3_5_1*
GSM466734	SRR029345	H1 cell line	H3K27me3	H1_H3K27me3_5_2
GSM466734	SRR029347	H1 cell line	H3K27me3	H1_H3K27me3_5_3
GSM466734	SRR029349	H1 cell line	H3K27me3	H1_H3K27me3_5_4*
GSM537683	SRR1068416	H1 cell line	H3K27me3	H1_H3K27me3_6_1
GSM605308	SRR067943	H1 cell line	H3K27me3	H1_H3K27me3_7_1
GSM409312	SRR018454	H1 cell line	H3K36me3	H1_H3K36me3_1_1
GSM428296	SRR018485	H1 cell line	H3K36me3	H1_H3K36me3_2_1
GSM428296	SRR018486	H1 cell line	H3K36me3	H1_H3K36me3_2_2
GSM433176	SRR020518	H1 cell line	H3K36me3	H1_H3K36me3_3_1
GSM450268	SRR026666	H1 cell line	H3K36me3	H1_H3K36me3_4_1
GSM450268	SRR026667	H1 cell line	H3K36me3	H1_H3K36me3_4_2
GSM466737	SRR029340	H1 cell line	H3K36me3	H1_H3K36me3_5_1
GSM466737	SRR029341	H1 cell line	H3K36me3	H1_H3K36me3_5_2
GSM466737	SRR029342	H1 cell line	H3K36me3	H1_H3K36me3_5_3
GSM466737	SRR029344	H1 cell line	H3K36me3	H1_H3K36me3_5_4
GSM466737	SRR029346	H1 cell line	H3K36me3	H1_H3K36me3_5_5
GSM537684	SRR1068417	H1 cell line	H3K36me3	H1_H3K36me3_6_1
GSM605309	SRR067944	H1 cell line	H3K36me3	H1_H3K36me3_7_1
GSM605317	SRR067952	H1 cell line	H3K56ac	H1_H3K56ac_1_1
GSM667627	SRR179741	H1 cell line	H3K56ac	H1_H3K56ac_2_1
GSM667627	SRR179742	H1 cell line	H3K56ac	H1_H3K56ac_2_2
GSM667627	SRR179743	H1 cell line	H3K56ac	H1_H3K56ac_2_3
GSM605318	SRR067953	H1 cell line	H3K79me1	H1_H3K79me1_1_1
GSM605319	SRR067954	H1 cell line	H3K79me1	H1_H3K79me1_2_1
GSM605320	SRR067955	H1 cell line	H3K79me1	H1_H3K79me1_3_1

GSM605321	SRR067956	H1 cell line	H3K79me2	H1_H3K79me2_1_1
GSM605322	SRR067957	H1 cell line	H3K79me2	H1_H3K79me2_2_1
GSM428289	SRR018482	H1 cell line	Input	H1_Input_1_1
GSM428289	SRR018483	H1 cell line	Input	H1_Input_1_2*
GSM428289	SRR018484	H1 cell line	Input	H1_Input_1_3
GSM433179	SRR020520	H1 cell line	Input	H1_Input_2_1
GSM450270	SRR026668	H1 cell line	Input	H1_Input_3_1*
GSM450270	SRR026669	H1 cell line	Input	H1_Input_3_2
GSM537682	SRR1068415	H1 cell line	Input	H1_Input_4_1
GSM605333	SRR067970	H1 cell line	Input	H1_Input_5_1
GSM605334	SRR067971	H1 cell line	Input	H1_Input_6_1
GSM605334	SRR067972	H1 cell line	Input	H1_Input_6_2
GSM605335	SRR067973	H1 cell line	Input	H1_Input_7_1
GSM605336	SRR067974	H1 cell line	Input	H1_Input_8_1
GSM605337	SRR067975	H1 cell line	Input	H1_Input_9_1
GSM605338	SRR067976	H1 cell line	Input	H1_Input_10_1
GSM667641	SRR097976	H1 cell line	Input	H1_Input_12_3
GSM605339	SRR067977	H1 cell line	Input	H1_Input_11_1
GSM667641	SRR097974	H1 cell line	Input	H1_Input_12_1
GSM667641	SRR097975	H1 cell line	Input	H1_Input_12_2
GSM667642	SRR179764	H1 cell line	Input	H1_Input_13_1
GSM667642	SRR179765	H1 cell line	Input	H1_Input_13_2

All datasets used in this study. The data marked by asterisk (*) were not considered for further analysis because the filtered reads are under the 15% of total reads.

ChIP-Seq, chromatin immunoprecipitation coupled with high-throughput DNA sequencing.
