# Supplementary material for: Comparative analysis of commonly used peak calling programs for ChIP-Seq analysis
Source: Genomics Inform. 2020 Dec 14;18(4):e42. doi: 10.5808/GI.2020.18.4.e42 (PMC7808876; doi:10.5808/GI.2020.18.4.e42)
Supplement: Supplementary Table 2. — Peak calling programs [file gi-2020-18-4-e42-suppl2.pdf]

Supplementary Table 2. Peak calling programs

Program	Tag density profile	Peak definition	Significance
CisGenome	Window sliding	Peak height and fold enrichment	FDR
MACS1	Window sliding	Peak height and fold enrichment	FDR
MACS2	Window sliding	Peak height and fold enrichment	FDR
MACS2 with a broad option	Window sliding	Peak height and fold enrichment	FDR
PeakSeq	Tag clustering	Peak height and fold enrichment	Compare to statistical model fitted with control data
SISSRs	Window sliding	Strand specific	Compare to normalized control data

FDR, false discovery rate.
