# Supplementary material for: Comparative analysis of commonly used peak calling programs for ChIP-Seq analysis
Source: Genomics Inform. 2020 Dec 14;18(4):e42. doi: 10.5808/GI.2020.18.4.e42 (PMC7808876; doi:10.5808/GI.2020.18.4.e42)
Supplement: Supplementary Table 3. — Alignment summary [file gi-2020-18-4-e42-suppl3.pdf]

Supplementary Table 3. Alignment summary

ID	Raw read number	Filtered read number	Mapped read number	Read mappability (%)
H3K4ac	93,867,431	60,825,283	40,724,497	66.95
H3K4me1	97,597,529	69,487,024	54,809,137	78.88
H3K4me2	35,818,415	21,537,301	15,588,642	72.38
H3K4me3	74,483,669	44,153,633	32,247,743	73.04
H3K9ac	88,210,929	64,599,746	43,785,656	67.78
H3K9me3	178,255,449	86,491,766	38,058,506	44.00
H3K27ac	26,900,401	16,587,444	11,191,010	67.47
H3K27me3	115,655,373	78,672,196	50,581,126	64.29
H3K36me3	192,914,242	104,342,495	66,812,765	64.03
H3K56ac	65,899,301	49,200,075	30,618,501	62.23
H3K79me1	57,832,894	41,461,437	27,895,622	67.28
H3K79me2	74,911,462	48,992,031	32,363,639	66.06
Input	302,343,443	192,111,851	132,198,714	68.81
