# Supplementary material for: Comparative analysis of commonly used peak calling programs for ChIP-Seq analysis
Source: Genomics Inform. 2020 Dec 14;18(4):e42. doi: 10.5808/GI.2020.18.4.e42 (PMC7808876; doi:10.5808/GI.2020.18.4.e42)
Supplement: Supplementary Table 4. — Average peak length (bp) [file gi-2020-18-4-e42-suppl4.pdf]

Supplementary Table 4. Average peak length (bp)

		MACS1	MACS2_def	MACS2_broad	PeakSeq	SISSRs	CisGenome
Point source	H3K4me2	970.7	385.8	1,005.0	1,245.1	57.7	662.9
	H3K4me3	1,941.8	1,018.0	1,848.1	1,596.8	58.4	1,209.2
	H3K9ac	1,490.4	498.9	955.5	1,188.9	89.5	555.5
	H3K27ac	1,017.1	368.1	942.4	847.6	125.0	537.8
Mixed source	H3K4ac	171.2	166.8	417.8	1,058.6	68.5	159.8
	H3K56ac	168.1	225.9	555.7	635.0	95.4	203.6
	H3K79me1	145.0	229.4	851.5	644.3	69.0	196.4
Broad source	H3K4me1	1,093.0	377.5	1,207.6	1,068.3	78.1	209.1
	H3K9me3	267.4	234.8	939.1	303.0	104.6	288.1
	H3K27me3	1,658.5	598.5	1,317.5	1,478.4	81.8	823.9
	H3K36me3	1,326.0	330.4	1,488.2	722.1	93.4	340.5
	H3K79me2	248.7	219.2	629.7	717.6	88.3	272.6
